# Designing a Graphene Metasurface Organic Material Sensor for Detection of Organic Compounds in Wastewater

**DOI:** 10.3390/bios13080759

**Published:** 2023-07-26

**Authors:** Khaled Aliqab, Jacob Wekalao, Meshari Alsharari, Ammar Armghan, Dhruvik Agravat, Shobhit K. Patel

**Affiliations:** 1 Department of Electrical Engineering, College of Engineering, Jouf University, Sakaka 72388, Saudi Arabia; 2 Department of Physics, Marwadi University, Rajkot 360003, India; 3 Department of Computer Engineering, Marwadi University, Rajkot 360003, India

**Keywords:** wastewater management, organic materials, sensor, mineral water sensor, biosensor

## Abstract

In many fields, such as environmental monitoring, food safety, and medical diagnostics, the identification of organic compounds is essential. It is crucial to create exceptionally sensitive and selective sensors for the detection of organic compounds in order to safeguard the environment and human health. Due to its outstanding electrical, mechanical, and chemical characteristics, the two-dimensional carbon substance graphene has recently attracted much attention for use in sensing applications. The purpose of this research is to create an organic material sensor made from graphene for the detection of organic substances like phenol, ethanol, methanol, chloroform, etc. Due to its high surface-to-volume ratio and potent interactions with organic molecules, graphene improves the sensor’s performance while the metasurface structure enables the design of highly sensitive and selective sensing elements. The suggested sensor is highly sensitive and accurate at detecting a broad spectrum of organic molecules, making it appropriate for a number of applications. The creation of this sensor has the potential to have a substantial impact on the field of organic sensing and increase the safety of food, medicine, and the environment. The graphene metasurface organic material sensor (GMOMS) was categorized into three types denoted as GMOMS1, GMOMS2, and GMOMS3 based on the specific application of the graphene chemical potential (GCP). In GMOMS1, GCP was applied on both the CSRR and CS surfaces. In GMOMS2, GCP was applied to the CS surface and the surrounding outer region of the CSRR. In GMOMS3, GCP was applied to the CSRR and the surrounding outer region of the CSRR surface. The results show that all three designs exhibit high relative sensitivity, with the maximum values ranging from 227 GHz/RIU achieved by GMOMS1 to 4318 GHz/RIU achieved by GMOMS3. The FOM values achieved for all the designs range from 2.038 RIU^−1^ achieved by GMOMS2 to 31.52 RIU^−1^ achieved by GMOMS3, which is considered ideal in this paper.

## 1. Introduction

Organic compound detection is an essential task in many fields, including environmental monitoring, food safety, and medical diagnostics [1]. Because of its exceptional electrical, mechanical, and chemical capabilities, graphene has recently become recognized as a viable material for sensing applications [2,3]. For the detection of various organic chemicals, such as gas and liquid-phase volatile organic compounds (VOCs), insecticides, and biomolecules, graphene-based sensors have been created [4]. High-performance sensors for the detection of gas-phase VOCs have been created using graphene [5,6,7]. The VOC molecules interact with the graphene surface to change the material’s electrical conductivity, which serves as the basis for the sensing mechanism [8]. A graphene-based sensor for the detection of ammonia was reported by Wang et al. [9], which showed good sensitivity to varying ammonia concentrations at room temperature, ranging from 10 to 50 ppm. Additionally, graphene-based sensors for the detection of liquid-phase organic compounds have been created [10], The adsorption of the organic molecules onto the graphene surface, which results in variations in the electrical conductivity of graphene, provides the basis for the sensing process [11,12]. A graphene-based sensor for the detection of 2,4,6-trinitrotoluene (TNT) in water was created by Hughes et al. [13]. The development of sensors for the detection of biomolecules has also utilized graphene. The precise binding of the biomolecules to the functionalized graphene surface, which results in changes in the electrical conductivity of graphene, is the basis for the sensing mechanism. A graphene-based biosensor for the detection of cardiac troponin I (cTnI), a biomarker for myocardial infarction, was described by Zhang et al. [14]. In the intervals of 0.3 pg/mL to 0.2 ng/mL, the sensor showed linear cTnl detection. The design of the sensing components can be highly flexible with the metasurface structure, which can increase the sensor’s sensitivity and selectivity toward organic molecules [15].

Advancements in sensing technology have brought THz research to the forefront. Significant progress has been made in areas such as THz imaging, THz-TDS advancement, and the creation of high-power THz through nonlinear effects [16,17]. Researchers have created new signal processing techniques, increasing the clarity and precision of terahertz waves and enabling more precise scanning and processing [18,19]. Considerable strides have been made in the creation of terahertz detectors. Scientists have achieved substantial advancements in terahertz detector flexibility and efficiency. Notable developments include a reduction in size owing to improvements in microfabrication methods, enabling them to be more compact and usable for a broader range of purposes [20,21]. Terahertz detectors are now used in more industries, such as communications, antennas, defense, and integrated circuits, because of their increased efficiency and adaptability [22]. Terahertz sensing has advanced significantly over the last two decades, beginning around 1980 with the advancement of THz time domain spectrometry [23,24]. Since then, many factors have changed. The result from THz-TDS by the formerly picometrix was the first step in initiating flexible systems based on terahertz waves [25]. Many previously unachievable investigations, such as those incorporating dispersion, are now feasible because of this development [26]. Contemporary terahertz science studies have increased as several groups have reported exciting advancements in the research of nanostructured materials. Determination of plasmon transmission induced at the tip in one graphene layer [27] began widespread motivation in the THz nanoscopic investigation of layered materials. Various terahertz generators, including intermittent and consistent sources, have attracted the attention of researchers [28]. The first revelation of pump-probe demonstrations using an aperture-less s-SNOM, as seen in [29], generated motivation in investigating THz photo physics with both femtosecond quick resolution and nanometer spatial precision, as noted in [30,31]. Further developments in these experiments have also been described and reported. Gallium nitride and gallium arsenide-based FET, FINFETs, SOI, Si MOS, and HEMTs arrays recently have shown effective terahertz sensing using a novel technique termed plasma wave circuitry. This development in THz digitalization holds the potential for broadening the uses of terahertz technology [32]. Wenfeng Fu and coauthors investigated organic compounds using a THz EIT metasurface sensor, achieving great response [33]. Yi Ma and coauthors studied an electrochemical sensor with a graphene layer and rod shape resonator structure, which is applicable for the detection of glucose [34]. Zeyu Wang and coauthors simulated a sensor used to remove an organic solvent from wastewater. This sensor was studied experimentally and compared to other techniques. From this study, it was observed that the simulation generated data that were close to the experimental data, thus validating the simulation method [35]. Peng Xiaoqian and coauthors studied rare earth elements that are present in wastewater and designed a sensor to remove those elements from water [36]. Chengbao Geng and coauthors studied a thin-film composite sensor for organic component detection and achieved 80.8% efficiency [37]. Jing Li and coauthors designed sensors for the recovery of NaCl from wastewater and achieved a 99.12% recovery rate [38].

A metasurface made from graphene consists of a vast collection of individual graphene cells that collectively exhibit plasmonic behavior when stimulated, resulting in exceptional electromagnetic properties [39]. These metasurfaces can be used to control the behavior of light, sound, and other electromagnetic waves in unusual ways, making them useful for various applications, including sensor design. One application of graphene meta-surfaces in sensor design is the development of optical sensors. The unique properties of graphene metasurfaces can be used to enhance the interaction between light and matter, leading to highly sensitive and accurate sensors [40]. Graphene metasurfaces also have the advantage of being highly tunable, meaning their properties can be adjusted to suit a specific application; this makes them useful for sensor design, where optimizing the sensor for a particular case of use is often necessary. Overall, graphene metasurfaces offer a wide range of possibilities for sensor design.

Organic chemicals are abundant in the environment and have the potential to be bad for both the ecosystem and human health. Therefore, the development of organic chemical detection sensors is essential for monitoring and controlling organic chemical concentrations. Due to their exceptional sensitivity and selectivity, graphene-based sensors have recently come to be recognized as a promising technology. In this study, we propose a highly sensitive and selective graphene metasurface organic material sensor for the detection of organic molecules. Our strategy includes combining plasmonic nanostructures with two-dimensional crystals to produce a highly sensitive and focused sensor platform for the detection of organic molecules.

## 2. Modeling and Design

**Figure 1:**
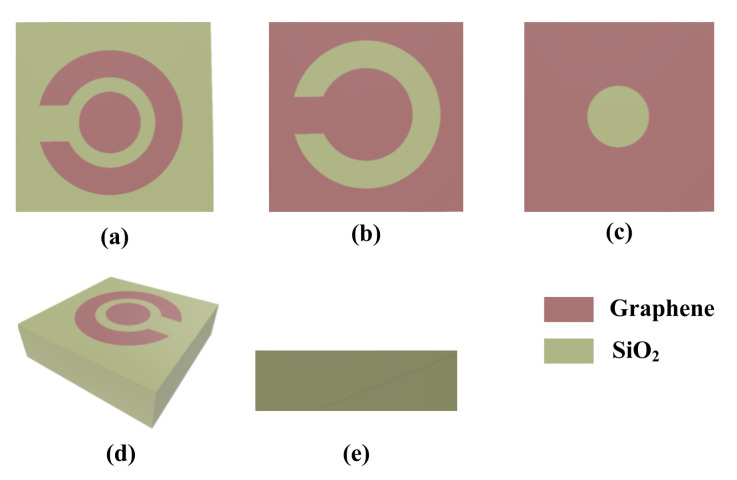
Proposed GMOMS for detecting organic compounds in wastewater: (**a**) top view of the GMOMS; (**b**) top view of the circular spring ring resonator (CSRR); (**c**) top view of the circular structure (CS); (**d**) 3D view of the optimized structure GMOMS; (**e**) side view and parameter of the GMOMS.

We proposed the design of a graphene metasurface organic material sensor (GMOMS) for the detection of organic compounds in wastewater. The GMOMS sensor design uses a circular spring ring resonator (CSRR) with a gap and a circular structure (CS) to achieve high sensitivity. The CSRR and CS combination act as a resonator that vibrates at a specific frequency when organic compound molecules are present, and the gap between the CSRR and CS allows molecules to pass through, increasing the sensor’s sensitivity. The GMOMS achieves excellent sensitivity for identifying alcohol in breath specimens by using a circular split ring resonator (CSRR) with a gap and a circular structure (CS). The circular structure (CS) and the CSRR are placed around the center of the sensor. Molecules of an organic compound can travel through the space between the CSRR and CS, which increases the sensor’s ability to respond to the amount of alcohol present in breath tests. Figure 1a–e provide a visual representation of the GMOMS design from various angles. The top aspects of the sensor are shown in Figure 1a–c, showing the CSRR, CS, and the CSRR gap. In this perspective, the sensor’s symmetry—which is essential for obtaining optimal performance—is highlighted, highlighting the rigorous design choices made.

Figure 1d depicts a three-dimensional view of the proposed sensor, with the circular structure (CS) at the sensor’s core. The CSRR and CS. in combination are purposefully created to facilitate extremely accurate detection of organic materials from wastewater. The CSRR and CS are shown in their horizontal orientations in Figure 1e, which shows an overhead view of the GMOMS. The silicon substrate employed averages 1500 nm thick and measures 10,000 nm × 10,000 nm. The external and innermost radii of each of the circles that make up the CSRR are 3500 and 2500 nm, respectively, while the CSRR gap is 870 nm by 870 nm in size. The CS has a 2300 nm radius.

The graphene conductivity ($\sigma_{s}$) can be defined with the help of Equations (1)–(4) [41]:

(1) $$\varepsilon \;\left(\omega \right)=1+\frac{\sigma_{s}}{\varepsilon_{0}\omega \nabla }$$

(2) $$\sigma_{intra}=\frac{-je^{2}k_{B}T}{\pi \hbar^{2}\;\left(\omega -j2\Gamma \right)}\left(\frac{\mu_{c}}{k_{B}T}+2\;\ln\left(e^{\frac{\mu_{c}}{k_{B}T}}+1\right)\right)$$

(3) $$\sigma_{inetr}=\frac{-je^{2}}{4\pi \hbar \;}\ln\left(\frac{2\left|\mu_{c}\right|-\left(\omega -j2\Gamma \right)\hbar }{2\left|\mu_{c}\right|+\left(\omega -j2\Gamma \right)\hbar }\right)$$

(4) $$\sigma_{s}=\sigma_{intra}+\sigma_{inter}$$

where ε is permittivity (F/m); ε_0_ is the permittivity of free space; *ω* is an angular frequency (red/s); $\sigma_{inetr}$ is interbend conductivity (S/m) and $\sigma_{intra}$ is intraband conductivity (S/m); ∇ is the thickness of the graphene layer in nm; $k_{B}$ is Boltzmann’s constant; ℏ is reduced Plank’s constant; and *μ_c_* is graphene chemical potential (Joule/mole), which is calculated using $\mu_{c}=\hbar \nu_{F}\sqrt{\pi CV_{DC}\;/\;e}$.

### Refractive Indices (RIs) of Alcohol Samples

Detection of alcohol based on a refractive index using COMSOL involves simulating the behavior of light passing through a sample of alcohol and measuring its refractive index. It is directly proportional to the concentration of solute particles in the solvent. The index of refraction for alcohol is among its most critical biophysical characteristics mentioned in Table 1.

## 3. Results and Discussion

In this part, we provide optimum research on the graphene metasurface organic material sensor (GMOMS) design and an investigation of the effect of graphene chemical potential on transmittance in three different cases. In the first case, the GMOMS design was optimized by varying several parameters, such as the CSRR’s gap size and radius and the CS radius, to obtain the best design. In the second case, we investigated the effect of graphene chemical potential on transmittance in three different cases. We analyzed the transmittance plots of the sensor at different frequencies for nine various graphene chemical potential values. The results demonstrate the performance of the GMOMS in detecting organic compounds with high sensitivity and selectivity. Additionally, we present the graphs presenting the electric field confinement (EFC) of the proposed GMOMS for different frequencies for the best-performing design. The detection graphs for the three different cases provide insight into the performance of the first case of the sensor.

### 3.1. Optimization of Parameters

**Figure 2:**
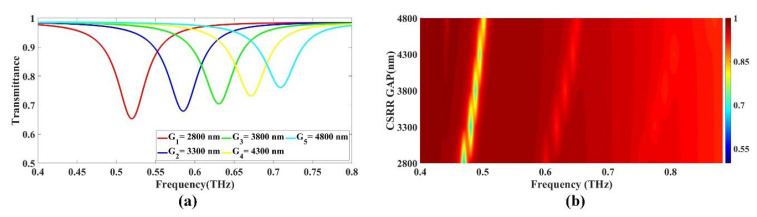
The effect of the CSRR gap on the transmittance response in an alcohol sensor design: (**a**) line; (**b**) color plots.

**Figure 3:**
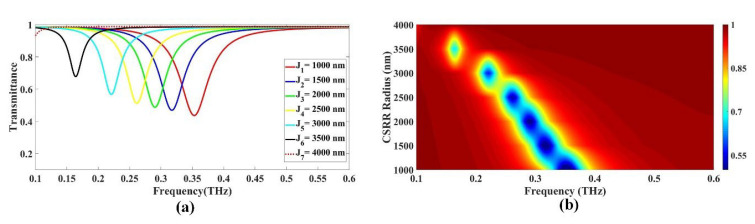
Investigating the influence of CSRR radius with minimal CS radius (R = 500 nm) on the transmittance response in alcohol sensor design: (**a**) line; (**b**) color plots.

**Figure 4:**
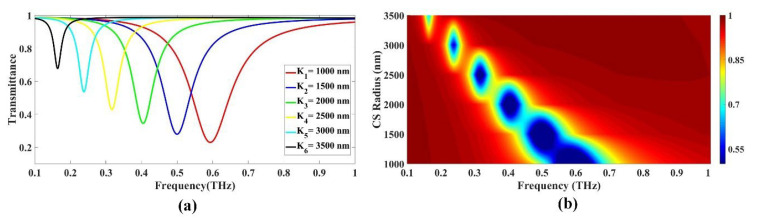
The effect of CS radius on the transmittance response in alcohol sensor design: (**a**) line; (**b**) color plots.

In order to optimize the design of the GMOMS, the size of the outer gap of the CSRR, the diameter of the CSRR, and the diameter of the CS were varied. The results from these variations are presented in Figure 2a,b, Figure 3a,b and Figure 4a,b. This study aimed to identify the most beneficial parameters to CSRR diameter and gap by exploring various design options.

#### 3.1.1. Variation in CSRR Gap

The study examined the effect of changing the gap of the CSRR on the transmittance drop, as shown in Figure 2a,b. The results show a shift in transmittance decrease to the right as the gap size grows. The gap length ranged from 2800 to 4800 nm. Figure 2a depicts the impact of increasing the gap size on the transmittance drop within the 0.4 to 1 THz range. To isolate the effect of the gap size, the diameter of the inner circle and other parameters were held constant throughout the study.

In Figure 2a, the relationship between the size of the gap and the corresponding values for transmittance drops as a function of frequency is presented. The gap is a structure that can be used to manipulate electromagnetic waves. The smallest gap size of 2800 nm exhibits a maximum transmittance drop of 0.652694 at a frequency of 0.52 THz. As the gap size increases to 3300 nm, the drop in transmittance decreases to 0.678889 at a frequency of 0.585 THz. Further, an increase in the gap size to 3800 nm results in a shift in transmittance to 0.704374 at a frequency of 0.631 THz. As the gap size increases to 4300 nm, the corresponding drop in transmittance is 0.731206 at a frequency of 0.672 THz. Further increase in gap size to 4800 nm results in a shift in transmittance to 0.760503 at a frequency of 0.709 THz, representing the lowest transmittance drop value, as shown in Figure 2a. The Fermi plot presented in Figure 2b clearly shows that the right-hand shift in transmittance occurs with an increase in the gap size.

#### 3.1.2. Variation in CSRR Radius

The transmittance as a function of frequency and the dimensions of the CSRR is demonstrated in Figure 3. From the graph presented in Figure 3a, it can be observed that the lowest drop in transmittance occurs at a radius of 1000 nm, with a value of 0.436313 and a frequency of 0.353 THz. As the radius increases to 1500 nm, the drop in transmittance decreases to 0.469054, with a frequency of 0.317 THz. As the radius of the CSRR further increases to 2000 nm, there is a decrease in transmittance, with a value of 0.487832 at a frequency of 0.29 THz. Moreover, when the radius increases to 2500 nm, the drop in transmittance is 0.512122 at a frequency of 0.262 THz. The drop in transmittance further increases to 0.567011 at a frequency of 0.221 THz, when the radius increases to 3000 nm. As the radius increases to 3500 nm, the drop in transmittance decreases to 0.683972 at a frequency of 0.663 THz. Finally, when the radius is increased to 3400 nm, there is a decrease in transmittance, with a value of 0.687059 at a frequency of 0.635 THz. The maximum radius of 3500 nm results in the lowest drop in transmittance of 0.677667, which occurs at a frequency of 0.164 THz, as shown in Figure 3(a). However, the minimal transmittance is achieved with the lowest radius value, R = 4000 nm, where the transmittance drops to 0.931552 at a frequency of 0.1 THz. The lowest radius of 1000 nm gives the optimal design in this case. The Fermi plot depicted in Figure 3b illustrates a left-hand shift in transmittance with increasing values for the radius.

#### 3.1.3. Variation in Radius of the CS

The impact of changing the radius of the CS on transmittance drop was investigated again in this study, and the results are shown in Figure 4a,b. The radius range tested was 1000 to 3500 nm, and the findings show that as the radius rose, the transmittance declined to the left.

Figure 4a explicitly shows the effect of increasing the radius on the transmittance drop within the frequency range 0.1 to 1 THz. In order to isolate the effect of radius from other parameters, the study kept the gap size and other variables constant. Figure 4a illustrates the relationship between CS radius and the corresponding transmittance drop values as a function of frequency. The lowest radius of 1000 nm exhibits a maximum transmittance drop of 0.229794 at a frequency of 0.593 THz. As the radius increases to 1500 nm, the drop in transmittance decreases to 0.279264 at a frequency of 0.5 THz. Further, an increase in the radius to 2000 nm results in a shift in transmittance to 0.345166 at a frequency of 0.405 THz. As the radius increases to 2500 nm, the corresponding transmittance drop is 0.42839 at a frequency of 0.317 THz. An increase in the radius to 3000 nm results in a shift in transmittance to 0.536134 at a frequency of 0.238 THz. An increase in the radius to 3500 nm results in a shift in transmittance to 0.677289 at a frequency of 0.164 THz, representing the lowest value for transmittance drop, as depicted in Figure 4a. The Fermi plot presented in Figure 4b shows the left shift in transmittance with an increase in CS radius.

#### 3.1.4. Variation in GCP

**Figure 5:**
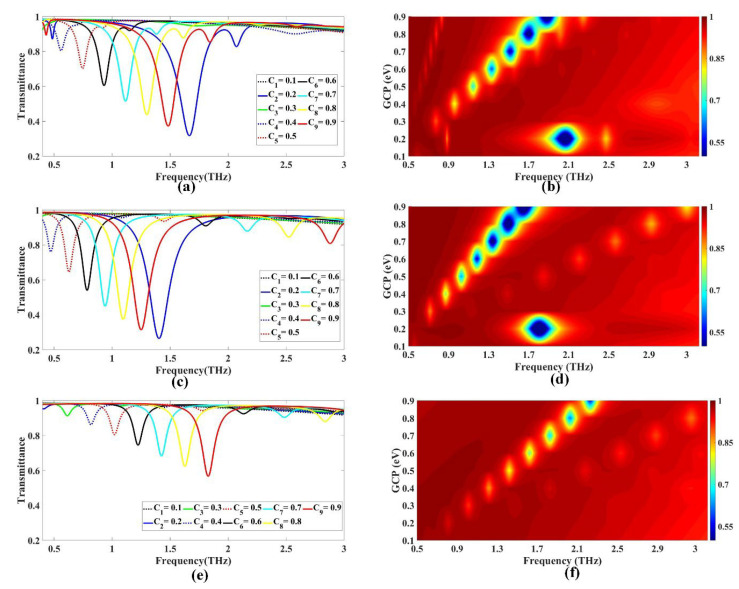
Investigating the influence of Fermi energy variation on the transmittance response for the different cases of the designed sensor: (**a**,**b**) represent the main plot and Fermi plot for the first case, which is the GMOMS1 sensor; (**c**,**d**) represent the main plot and Fermi plot for the second case, which is the GMOMS2 sensor; (**e**,**f**) represent the main plot and Fermi plot for the third case, which is the GMOMS3 sensor.

**Figure 6:**
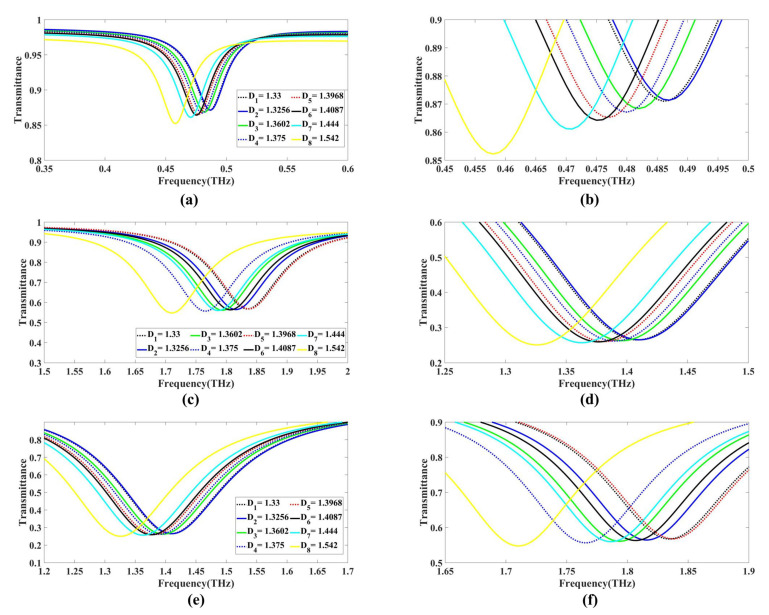
Graph of the three main planned GMOMS instances used for detection: (**a**,**b**) depict detection plots for GMOMS1; (**c**,**d**) demonstrate the detection plots for GMOMS2; (**e**,**f**) illustrate the detection plots for GMOMS3.

The sensor was divided into three cases, denoted as GMOMS1, GMOMS2, and GMOMS3, depending on the specific application of GCP. In the first case (GMOMS1), GCP was applied to both the CSRR and CS surfaces, and the results are presented in Figure 5a,b. Similarly, the second case (GMOMS2) involved the application of GCP to the CS surface and the outer region surrounding the CSRR, excluding the CSRR itself, with the results shown in Figure 5c,d. Finally, the third case (GMOMS3) involved applying GCP to the CSRR and the outer region surrounding the CSRR surface, excluding the CS surface, with results shown in Figure 5e,f. This section provides a comprehensive analysis of the sensor response in each of the three cases in terms of GCP variation. Figure 5a–f displays the transmittance plots for the graphene metasurface organic compound sensor. These figures show the sensor’s transmittance at different frequencies for three different situations, at nine different graphene chemical potential (GCP) values ranging from 0.1 to 0.9 eV in 0.1 eV increments. When GCP was applied to both the CSRR and CS surfaces in the first example, the transmittance decreases were found to move to the right as the GCP value grew, as shown in Figure 5a. The greatest drop in transmittance occurred at a GCP of 0.2 eV, while the best transmittance was obtained when GCP was equal to 0.2 eV. The transmittance value at a frequency of 1.666 THz decreased to 0.317 at this GCP value. The Fermi plot in Figure 6b shows the rightward shift in transmittance as GCP values increase. In the second case, where GCP was applied to the CS surface and the outer region surrounding the CSRR, excluding the CSRR, the transmittance values were observed to decrease as the GCP value increased, and the maximum drop in transmittance was observed at GCP = 0.2 eV, as illustrated in Figure 5c. At this GCP value, the transmittance value decreased to 0.264 at a frequency of 1.404 THz. Figure 5d presents the Fermi plot indicating the rightward shift in transmittance with increasing GCP values. In the third case, where GCP was applied to the CSRR and the outer region surrounding the CSRR surface, excluding the CS surface, the transmittance values were observed to decrease as the GCP value increased. At a GCP value of 0.2 eV, the transmittance value decreased to 0.924 at 3 THz frequency. The maximum transmittance drop occurs at a GCP value of 0.9 eV, where transmittance decreases to 0.567 at a frequency of 1.828 THz. Thus, the optimal transmittance is achieved at a GCP value of 0.9 eV, as shown in Figure 5e. Figure 5f presents the Fermi plot indicating the rightward shift in transmittance with increasing GCP values.

Overall, we can see that the transmittance values obtained in the three different cases varied significantly, with the optimal transmittance achieved at different GCP values in each case. These results suggest that the choice of GCP value has a significant impact on the performance of the sensor, and consequently, careful consideration should be given while selecting an appropriate GCP value depending on the specific application requirements.

### 3.2. Detection

Figure 6a–e present the detection results for the three different cases of GMOMS (GMOMS1, GMOMS2, and GMOMS3) in terms of transmittance dips and their implications for detection. For GMOMS1 (case 1), Figure 6a shows a graph indicating specific frequencies at which the transmittance dips occur, suggesting their effectiveness for detection.

The zoomed-in area of the picture in Figure 6b likewise shows a decrease in transmittance between 0.45 and 0.5 THz, demonstrating the design’s sensitivity to these frequencies. Therefore, the appropriate frequency range selection is crucial to achieve efficient detection, making these detection graphs valuable in comprehending the design’s performance. For GMOMS2 (case 2), Figure 6c,d depict the trend in transmittance dips and the frequencies displaying the most significant drops in transmittance, indicating their potential for detection. The zoomed-in section of the graph in Figure 1d reveals a decrease in transmittance within the 0.2 to 1.55 THz frequency range, highlighting the design’s sensitivity to these frequencies. Hence, selecting the appropriate frequency range is vital to achieving optimal detection performance, making these detection graphs valuable for understanding the design’s effectiveness. For GMOMS3 (case 3), Figure 6e,f display the trend in transmittance dips, emphasizing the frequencies that show the most significant drops in transmittance, suggesting their potential for detection. The zoomed-in portion of the graph in Figure 6e shows a decline in transmittance within the 1.6 to 2 THz frequency range, underscoring the design’s sensitivity to these frequencies. These results demonstrate the importance of carefully selecting the appropriate frequency range for achieving optimal detection performance. Therefore, the detection graphs presented in this figure offer valuable insights into the effectiveness of the GMOMS3 design.

### 3.3. Electric Field Analysis for the Best-Performing Design (GMOMS3)

**Figure 7:**
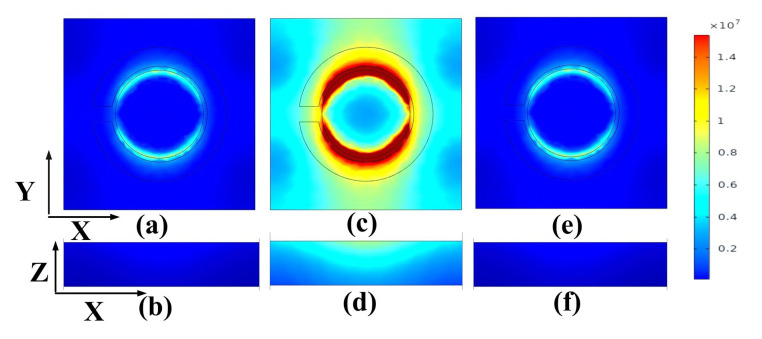
Electric field concentration (EFC) plots for the proposed GMOMS3 at different frequencies: (**a**,**b**) 0.475 THz; (**c**,**d**) 0.46 THz; (**e**,**f**) 0.49 THz.

Figure 7 illustrates the EFC of the proposed GMOMS3 for various frequencies and was used to analyze the GMOMS3′s transmittance performance. The electric field intensities for the XY and XZ graphs are displayed in Figure 7a–f. At 0.46 and 0.49 THz, Figure 7a,b,e,f show excellent transmittance, as demonstrated by the low scattering of the EFC on the GMOMS3. This indicates lower absorption and higher transmittance, which aligns with our findings. However, at 0.475 THz, Figure 7c,d show a drop in transmittance, as indicated by the high EFC on the metasurface structure, validating the dip in transmittance response.

We can calculate the sensitivity (*S*) of the proposed sensor by using Equation (5):

(5) $$S=\frac{\Delta \lambda }{\begin{array}{c} \Delta n \end{array}}$$

where *λ* is wavelength and *n* is the refractive index of the materials. The figure of merit can be calculated with the given relation:

(6) $$FoM=\frac{\Delta \lambda }{\lambda_{\begin{array}{c} FWHM \end{array}}}$$

where the unit of *FoM* is RIU^−1^. For the detection limit DL, the relation with *S* and *Q* is

(7) $$\mathrm{DL}=\frac{\lambda }{\begin{array}{c} 20SQ \end{array}}$$

where *Q* is a quality factor and calculated by taking the ratio of central wavelength and wavelength at FWHM of the defect mode, and the unit of DL is RIU. The performance parameters are compared for three cases in Table 2.

For the first case, the highest relative sensitivity achieved by this sensor is 227 GHz/RIU, demonstrating its capability to detect changes in the refractive index accurately. The sensor has an exceptional capacity to distinguish between different refractive indices, as evidenced by its figure of merit (FOM) values, which range from 3.062 to 6.313 RIU^−1^. The quality factor is a crucial parameter that characterizes the sharpness in the transmittance drop. The range of Q values is 12.052 to 13.528, indicating its high precision in detecting even minor variations in transmittance. The maximum value of detection accuracy is 27.778 s. The proposed sensor exhibits exceptional resolution, as evidenced by the lowest FWHM value of 0.036 THz. The sensor’s resolution is evaluated, and the highest and lowest values are 0.058 and 0.033, respectively. The sensor also demonstrates a detection limit of 0.250 and a dynamic range with the highest value of 2.567. For the second case (GMOMS2), the sensor demonstrates a maximum sensitivity of 455 GHz/RIU, significantly higher than the sensitivity of GMOMS1. Moreover, the figure of merit (FOM) values obtained by the GMOMS2 sensor range between 1.514 and 2.038 RIU^−1^. These values are considerably lower in contrast to the FOM values for GMSOM1. Therefore, it can distinguish refractive indices less effectively than the GMOMS1 sensor. Furthermore, in comparison to GMOMS1, the quality factor values for this sensor are low, suggesting that it has less accuracy in detecting transmittance drops than GMOMS1. This sensor’s range of Q values is from 5.973 to 6.322 which are lower in comparison to GMOMS1′s Q values. This sensor’s accuracy in detecting sudden drops in transmittance is low, with a maximum value of 4.505, which is lower than that of GMOMS1. Moreover, on the other hand, the lowest value for FWHM for the proposed sensor is 0.222 THz, which is higher than that for GMOMS1; this indicates that the proposed sensor has a higher resolution than GMOMS1. Furthermore, the highest detection limit for this sensor is 1.063, which is significantly higher than that for GMOMS1. The highest value for the dynamic range for the proposed sensor is 2.986, which is higher than that for GMOMS1.

Compared to the GMOMS1 and GMOMS2 sensors, the GMOMS3 sensor demonstrates a significantly higher sensitivity at 4318 GHz/RIU. The figures of merit (FOM) achieved range from 4.407 −31.520 RIU^−1^, notably higher than those of the GMOMS1 and GMOMS2 sensors. In comparison to the GMOMS2 sensor, the GMOMS3 sensor exhibits a quality factor (Q) ranging from 12.761 to 13.409, indicating superior performance over the GMOMS2 sensor, but not as high as the GMOMS1 sensor design. The sensor offers a significantly higher sensor resolution, ranging from 0.103 to 0.150, compared to the GMOMS1, but slightly less than the GMOMS1 sensor design. The sensor’s detection limit ranges from 0.071 to 0.241, indicating its ability to detect low concentrations of alcohol, although this is lower than that for the GMOMS1 and GMOMS2 sensors. The sensor exhibits the lowest value for FWHM, 0.134 THz, higher than that for the GMSOM1 and lower than the GMOMS2 sensor. It also achieves a maximum dynamic range of 4.906, significantly higher than that of the GMOMS1 and GMOMS2 sensor designs. The uncertainty ranges from 0.007 to 0.019, which is higher than that for the GMSOM1 and GMOMS2 sensors. The maximum detection accuracy achieved is 7.463, which is lower than that for the GMOMS1, but greater than the detection accuracy for the GMOMS2 sensor designs.

### 3.4. Encoding for the GMOMS Sensor

The transmittance response of the proposed structure was analyzed at different values for GCP, resulting in the realization of an NOR gate. Specifically, when k_1_ = 0.1 eV and k_2_ = 0.1 eV, a perfect transmittance of 1 was achieved. On the other hand, when k_1_ = 0.1 eV and k_2_ = 0.9 eV or when k_1_ = 0.9 eV and k_2_ = 0.1 eV or when k_1_ = 0.9 eV and k_2_ = 0.9 eV, a drop in transmittance, considered as 0, was observed.

**Figure 8:**
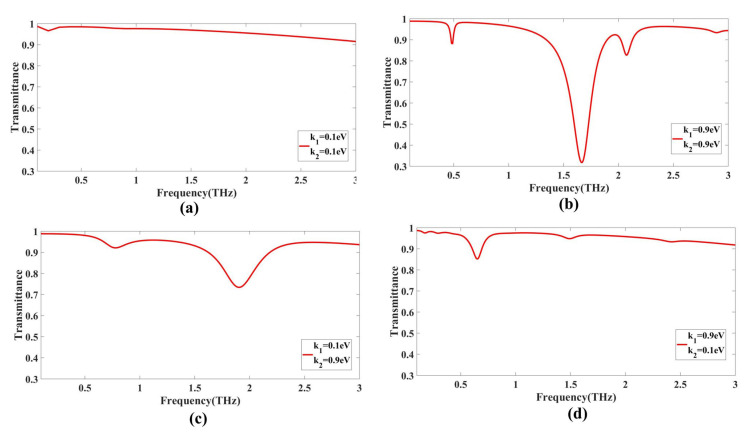
Transmittance response from the GMOMS sensor: (**a**) outcome of GMOMS when k_1_ = 0.1 eV and k_2_ = 0.1 eV; (**b**) outcome of GMOMS when k_1_ = 0.9 eV and k_2_ = 0.9 eV; (**c**) outcome of GMOMS when k_1_ = 0.1 eV and k_2_ = 0.9 eV; (**d**) outcome of GMOMS when k_1_ = 0.9 eV and k_2_ = 0.1 eV.

In the first case, a magnitude of 0.95 was obtained at a frequency of 0.1 THz. Here, the simulation focused on an electromagnetic system where frequencies in the terahertz range were used in this domain. The magnitude of 0.95 obtained refers to the amplitude of an electromagnetic field at a particular point in the system. The field could be electric or magnetic, and its amplitude may be expressed in units such as volts per meter (V/m) or tesla (T), depending on the specific simulation. 6.9. Similarly, in case 2, a magnitude of 0.9 was obtained at a frequency of 1.8 THz. In case 3, a magnitude of 0.96 was obtained at a frequency of 0.3 THz. For case 4, a magnitude of 0.9 is obtained at a frequency of 1.9 THz, as illustrated in Figure 8.

### 3.5. Comparison of the Sensor with Other Similar Works

Furthermore, a comprehensive comparison of various cases of the GMOMS sensor with other similar works is considered. The comparison was carried out based on several key performance parameters, including S (sensitivity), FOM (figure of merit), DL (detection limit), and Q (quality factor). These parameters are crucial in evaluating the effectiveness and efficiency of the GMOMS sensor in comparison to other relevant works. The results from this comparison are summarized in Table 3. The results reveal the GMOMS sensor’s performance and how it compares to other similar works in terms of the aforementioned parameters.

## 4. Conclusions

In this paper, three designs exhibit high relative sensitivity, with the maximum values ranging from 227 GHz/RIU achieved by GMOMS1 to 4318 GHz/RIU achieved by GMOMS3. The FOM values achieved by all of the designs range from 2.038 RIU^−1^ achieved by GMOMS2 to 31.52 RIU^−1^ achieved by GMOMS3, which are considered ideal in this case. The excellent electrical conductivity, high surface area, and strong mechanical properties of graphene make it an ideal material for sensing applications. The graphene metasurface breath-alcohol sensor is designed to detect the presence of alcohol in exhaled breath. Alcohol molecules interacting with the graphene metasurface produce a change in the conductivity of the graphene. The sensor measures this change in conductivity, providing an accurate measurement of the organic compound concentration in wastewater. The GMOMS sensor has several advantages over traditional organic compound analyzers. Its high sensitivity enables it to detect very low concentrations of organic compounds in wastewater. The GMOMS sensor is also low-cost and has a compact size, making it suitable for use in various settings where space is limited. The sensor is reusable, further reducing the cost of organic compound detection. Overall, developing the GMOMS sensor represents a significant advancement in organic compound detection and demonstrates the potential for two-dimensional crystals in developing high-performance sensors. Future research can focus on improving the sensitivity and selectivity of the sensor by optimizing its design and exploring the use of different two-dimensional crystals.

## Figures and Tables

**Table 1:** Samples from alcohol samples and their respective RIs [42].

Sample	RI (n)
methanol	1.33
acetone	1.3256
propanol	1.3602
ethanol	1.375
butanol	1.3968
pentanol	1.4087
chloroform	1.444
phenol	1.542

**Table 2:** The performance parameters for GMOMS for the three cases (GMOMS1, GMOMS2, GMOMS3).

**GMOMS1**	f(THz)	0.486	0.487	0.482	0.48	0.477	0.475	0.471	0.458
	n	1.33	1.3256	1.3602	1.375	1.3968	1.4087	1.444	1.542
	df		0.001	0.005	0.002	0.003	0.002	0.004	0.013
	dn		0.0044	0.0346	0.0148	0.0218	0.0119	0.0353	0.098
	S(GHz/RIU)		227	145	135	136	168	113	133
	FWHM(THz)	0.037	0.036	0.037	0.036	0.037	0.037	0.037	0.038
	FOM(RIU-1)		6.313	3.906	3.754	3.719	4.542	3.062	3.491
	Q		13.528	13.027	13.333	12.891	12.837	12.729	12.052
	DL		0.2587	0.282	0.366	0.334	0.304	0.38	0.25
	DR		2.567	2.506	2.53	2.46	2.45	2.447	2.349
	DA		27.778	27.027	27.778	27.027	27.027	27.027	26.316
	SR		0.058	0.041	0.049	0.046	0.051	0.043	0.033
**GMOMS2**	f(THz)	1.408	1.41	1.396	1.39	1.381	1.377	1.363	1.326
	n	1.33	1.3256	1.3602	1.375	1.3968	1.4087	1.444	1.542
	df		0.002	0.014	0.006	0.009	0.004	0.014	0.037
	dn		0.0044	0.0346	0.0148	0.0218	0.0119	0.0353	0.098
	S(GHz/RIU)		455	405	405	413	336	397	378
	FWHM	0.222	0.223	0.222	0.223	0.222	0.222	0.222	0.222
	FOM(RIU-1)		2.038	1.823	1.818	1.86	1.514	1.786	1.701
	Q		6.322	6.288	6.233	6.221	6.203	6.14	5.973
	DL		1.063	0.73	0.905	0.8	1.202	0.745	0.614
	DR		2.986	2.963	2.944	2.931	2.922	2.893	2.814
	DA		4.484	4.505	4.484	4.505	4.504	4.505	4.505
	SR		0.483	0.295	0.367	0.33	0.404	0.295	0.232
**GMOMS3**	f(THz)	1.835	1.816	1.794	1.766	1.837	1.807	1.786	1.71
	n	1.33	1.3256	1.3602	1.375	1.3968	1.4087	1.444	1.542
	df		0.019	0.022	0.028	0.071	0.03	0.021	0.076
	dn		0.0044	0.0346	0.0148	0.0218	0.0119	0.0353	0.098
	S(GHz/RIU)		4318	635	1892	3257	2521	595	776
	FWHM	0.137	0.137	0.136	0.136	0.137	0.136	0.135	0.134
	FOM(RIU-1)		31.52	4.675	13.911	23.773	18.537	4.407	5.787
	Q		13.255	13.191	12.985	13.409	13.287	13.23	12.761
	DL		0.035	0.225	0.071	0.033	0.052	0.241	0.133
	DR		4.906	4.865	4.789	4.963	4.9	4.861	4.671
	DA		7.299	7.353	7.353	7.299	7.353	7.407	7.463
	SR		0.15	0.143	0.135	0.108	0.133	0.144	0.103

**Table 3:** The performance parameters of GMOMS for the three cases (GMOMS1, GMOMS2, GMOMS3).

Sensor	S (GHz/RIU)	FOM (RIU^−1^)	DL (RIU)	Q	Detection
GMOMS1	227	6.313	0.25	13.528	Detection of organic materials in wastewater
GMOMS2	455	2.038	0.614	6.322	
GMOMS3	4318	31.52	0.033	13.409	
Ref. [42]	500 nm/RIU	5000	10^−4^	5.34 × 10^3^	Detection of organic materials in wastewater
Ref. [43]	1000 nm/RIU	10^2^	10^−5^	6418.5	Organic compound sensing applications
Ref. [44]	144.369 nm/RIU	-	-	-	Detection of water concentration in ethanol
Ref. [45]	756 nm/RIU	593.9	-	1092	Detection of ethanol and methanol
Ref. [46]	2350 nm/RIU	195	-	-	Alcohol sensing application
Ref. [47]	240	-	-	-	Detection of protein
Ref. [48]	233	-	-	-	Detection of hemoglobin
Ref. [49]	207	3.86	0.17	13.11	Detection of cancer

## Data Availability

The data will be made available at a reasonable request to the corresponding author.
